# Three-Dimensionally Printed Ternary Composites of Polyamide: Effect of Gradient Structure on Dimensional Stability and Mechanical Properties

**DOI:** 10.3390/polym16192697

**Published:** 2024-09-24

**Authors:** Qiming Chen, Zewei Cai, Dhandapani Kuzhandaivel, Xianliang Lin, Jianlei Wang, Suyu Chen

**Affiliations:** 1 College of Chemistry, Fuzhou University, Fuzhou 350116, China; 2 CAS Key Laboratory of Design and Assembly of Functional Nanostructures, Fujian Key Laboratory of Nanomaterials, Fujian Institute of Research on the Structure of Matter, Chinese Academy of Sciences, Fuzhou 350002, China; 3 Fuzong Clinical Medical College of Fujian Medical University (The 900th Hospital of Joint Logistic Support Force), Fuzhou 350025, China; 4 Fujian Universities and Colleges Engineering Research Center of Modern Facility Agriculture, Fujian Polytechnic Normal University, Fuzhou 350300, China; 5 Fujian Science and Technology Innovation Laboratory for Optoelectronic Information of China, Fuzhou 350108, China; 6 Department of Endoscopy Center, Clinical Oncology School of Fujian Medical University, Fujian Cancer Hospital, Fujian Branch of Fudan University Shanghai Cancer Center, FuMa Rd. 420, Fuzhou 350014, China

**Keywords:** fused deposition modeling (FDM), ternary composites, polyamide 6 (PA6), dimensional stability, gradient structure

## Abstract

Fused deposition modeling (FDM) 3D printing has the advantages of a simple molding principle, convenient operation, and low cost, making it suitable for the production and fabrication of complex structural parts. Moving forward to mass production using 3D printing, the major hurdle to overcome is the achievement of high dimensional stability and adequate mechanical properties. In particular, engineering plastics require precise dimensional accuracy. In this study, we overcame the issues of FDM 3D printing in terms of ternary material compounds for polyamides with gradient structures. Using multi-walled carbon nanotubes (MWCNTs) and boron nitride (BN) as fillers, polyamide 6 (PA6)-based 3D-printed parts with high dimensional stability were prepared using a single-nozzle, two-component composite fused deposition modeling (FDM) 3D printing technology to construct a gradient structure. The ternary composites were characterized via DSC and XRD to determine the optimal crystallinity. The warpage and shrinkage of the printed samples were measured to ensure the dimensional properties. The mechanical properties were analyzed to determine the influence of the gradient structures on the composites. The experimental results show that the warpage of pure polymer 3D-printed parts is as high as 72.64%, and the introduction of a gradient structure can reduce the warpage to 3.40% by offsetting the shrinkage internal stress between layers. In addition, the tensile strength of the gradient material reaches up to 42.91 MPa, and the increasing filler content improves the interlayer bonding of the composites, with the bending strength reaching up to 60.91 MPa and the interlayer shear strength reaching up to 10.23 MPa. Therefore, gradient structure design can be used to produce PA6 3D-printed composites with high dimensional stability without sacrificing the mechanical properties of PA6 composites.

## 1. Introduction

Polyamide 6, also known as nylon 6, has been widely utilized in engineering plastics because of its exceptional mechanical properties and resilience to harsh physical and chemical environments. It has applications in various industries [1,2,3,4]. PA6 is widely recognized as an optimal material for replacing heavy metals in lightweight automotive technology owing to its exceptional mechanical strength, processing capabilities, high temperature resistance, abrasion resistance, and corrosion resistance [5,6]. Growing engineering requirements in the automotive industry have led to a significant increase in the demand for structurally complex lightweight components, resulting in the increased use of PA6. However, the traditional injection molding process used for processing and molding PA6is limited in its ability to produce complex due to the nature of the process method and difficulty in control. These restricts its application in the field of lightweight automotive technology and its ability to meet the growing demand for lightweight components [7].

Three-dimensional printing, a leading technology in material processing and manufacturing, offers extensive design freedom and customization. Its widespread adoption has led to significant changes in various industries, particularly the production of complex structural components [8,9]. FDM 3D printing, one of the most widely used technologies, is practical, cost-effective, and environmentally friendly. Its simple working principle, low equipment and maintenance costs, and pollution-free production process make it well-suited for producing complex structural parts [10]. Process parameters, such as temperature, speed, and material flow, significantly influence the quality and characteristics of the final printed object [11,12,13]. Additionally, the advent of multi-material FDM printing has expanded the potential for creating complex, high-performance parts, although it introduces complexities related to the material feeding mechanisms and interlayer adhesion [14]. Ongoing research and development aims to optimize the process parameters of FDM, expand its capabilities, address inherent limitations, and explore new applications in various industries [15,16].

**Figure 1:**
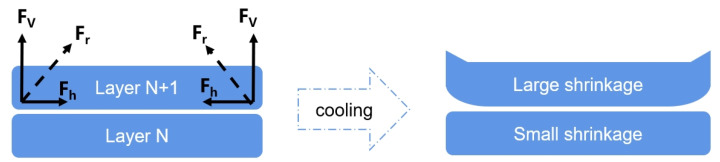
Schematic of the dimensional instability of the part resulting from FDM 3D printing.

With FDM 3D printing technology, the raw material undergoes a solid–liquid–solid phase transition during the printing process, where it is constructed layer by layer [17]. The lower layer exhibited a reduced temperature, which resulted in diminished molecular chain mobility and subsequent volume contraction. Conversely, the newly formed upper layer sustains an elevated temperature, thereby facilitating enhanced molecular chain movement and resulting in a pronounced volume shrinkage. The variation in the volume shrinkage rates between these layers induced internal shrinkage stresses (Fh and Fv). Throughout the printing process, these internal stresses (resultant Fr) accumulate, leading to deformations such as bending and delamination between layers, as depicted in Figure 1. Owing to the time difference in layer stacking, a temperature difference arises between the layers. The bottom deposition layer cooled rapidly upon contact with the printing platform, resulting in minimal shrinkage and slower molecular chain movement [18]. In contrast, the newly stacked layer experiences higher volume shrinkage and faster molecular chain movement owing to higher temperatures [19]. This variation in the volume reduction between the layers causes internal stress, which accumulates as the printing process continues, potentially leading to dimensional instability in the sample, such as bending, warping, and interlayer peeling [20]. The molecular chain arrangement is more orderly, and the intermolecular forces are stronger when the polymer crystallinity is higher, making the volume contraction inequality during cooling more noticeable and affecting the dimensional stability of the printed sample. PA6’s crystallinity makes it prone to warping, bending, and delamination during the 3D printing process, which can result in unsuitable printed products or even printing failures, thereby restricting its applications in the field [21].

The crystallinity of polyamide 6 (PA6) has been shown to influence its mechanical properties, which can affect the performance of 3D-printed components. In Sakai et al., the study found that an increase in the crystallinity of PA6, achieved through longer heat treatment times, made the matrix resin of carbon fiber-reinforced polyamide 6 (CFRTP) more brittle, which could potentially lead to warping and bending issues during the 3D printing process. However, the study also noted that while crystallinity was related to bending strength, it resulted in a decrease in the Young’s modulus, which is a measure of stiffness [19]. The mechanical properties and dimensional stability of PA6 during 3D printing are influenced by various factors, including the reinforcement with continuous carbon fibers and printing parameters [20].

Advances in fused deposition modeling (FDM) 3D printing technology have been significant in addressing both material and process limitations to expand applications. Notably, the development of polyamide-based composites to mitigate issues such as warping and distortion in pure polyamide prints has been highlighted [21]. Additionally, the mechanical properties of FDM prints have been improved through post-processing techniques such as ultrasonic strengthening, which has been shown to enhance the tensile strength and Young’s modulus of acrylonitrile butadiene styrene (ABS) samples [22]. While some studies focused on material enhancements, others emphasized the importance of the printing process itself. For instance, the material-feeding mechanism in multimaterial FDM printing is crucial for the interlayer adhesion quality, which determines the strength of the final product. Therefore, eliminating the internal stress caused by interlayer shrinkage during PA6 printing and improving the dimensional stability of printed products are crucial for expanding their applications in 3D printing.

In recent studies, researchers have enhanced PA6 by incorporating amorphous polymers and inorganic fillers to minimize the interlayer shrinkage and improve the dimensional stability of PA6 printed components. Jia et al. [23]. combined PA6 with maleic anhydride-grafted ethylene-octene copolymer (POE-g-MAH) and polypropylene (PS) to fabricate FDM 3D printing filaments. The addition of amorphous POE-g-MAH and PS disrupted the regular arrangement of PA6 molecular chains, reducing their crystallinity, and the molecular chains of the blends were irregularly arranged, significantly reducing warping in the PA6 composite printed products [23,24]. Furthermore, introducing functional fillers, such as graphene [25,26] and boron nitride [26], can further reduce warping and shrinkage, enhancing the mechanical, electrical, and thermal conductivities of the composites. However, it is often necessary to introduce a higher content of amorphous polymers (mass fraction greater than 40%) into PA6 to ensure its high dimensional stability during FDM printing, and the incorporation of amorphous polymers reduces the mechanical properties of the composites, thus seriously sacrificing the mechanical properties while enhancing the dimensional stability of PA6 for 3D printing.

To address the issues of dimensional stability of PA6 during 3D printing, this study employed single-nozzle two-component composite fused deposition modeling (S2-FDM) technology to create a gradient structure. The successful preparation of 3D-printable PA6 composite filaments with high filler content was achieved by incorporating multi-walled carbon nanotubes and boron nitride as dimensional stability modifiers. S2-FDM technology was utilized to precisely control the gradient structure, resulting in PA6 composites with high dimensional stability. The PA6 prints produced using this method exhibited exceptional mechanical properties and dimensional stability. This straightforward approach to building gradient structures through S2-FDM 3D printing presents a new solution for the challenge of warpage during PA6 printing.

## 2. Experiments

### 2.1. Materials

**Figure 2:**
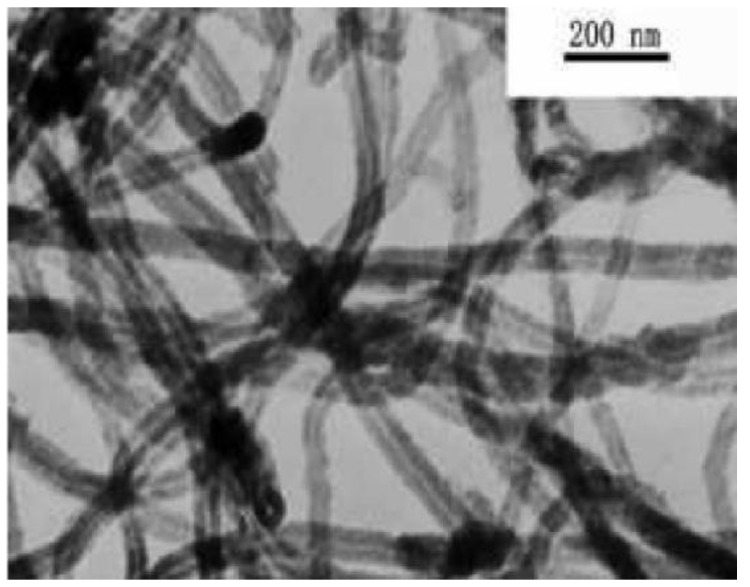
TEM photographs of carboxyl-purified multi-walled carbon nanotubes.

Polyamide 6 (PA6), model number TP-4208, was manufactured by Taiwan Jisheng Industrial Co., Ltd., located in Taiwan, China. The maleic anhydride-grafted ethylene-octene copolymer (POE-g-MAH), model number PC-28, was manufactured by Foshan Nanhai Park Chen Polymer New Material Co., Ltd., located in Foshan, China. The carboxylated multi-walled carbon nanotubes (MWCNTs-COOH) produced by Suzhou Carbon Fund Graphene Technology Co. have an outer diameter of 8–15 nm in OD and a length of 30–50 μm, and are manufactured in Suzhou, Jiangsu Province, China. TEM images are shown in Figure 2. The boron nitride (BN) with a particle diameter of about 25 μm was manufactured by Jinan Zhiding Welding Materials Co, located in Jinan, China

### 2.2. Preparation of PA6/POE-g-MAH Blended Strands

Initially, the PA6 and POE-g-MAH pellets were dried in an oven at 85 °C and 60 °C, respectively, for 12 h. After drying, the PA6 and POE-g-MAH were thoroughly mixed at weight ratios of 100:0, 95:5, 90:10, and 85:15. The mixed pellets were then melt-extruded using a twin-screw extruder to prepare strands with a diameter of 1.75 mm.

### 2.3. Preparation of High-Filler-Loaded Blended Strands

**Figure 3:**
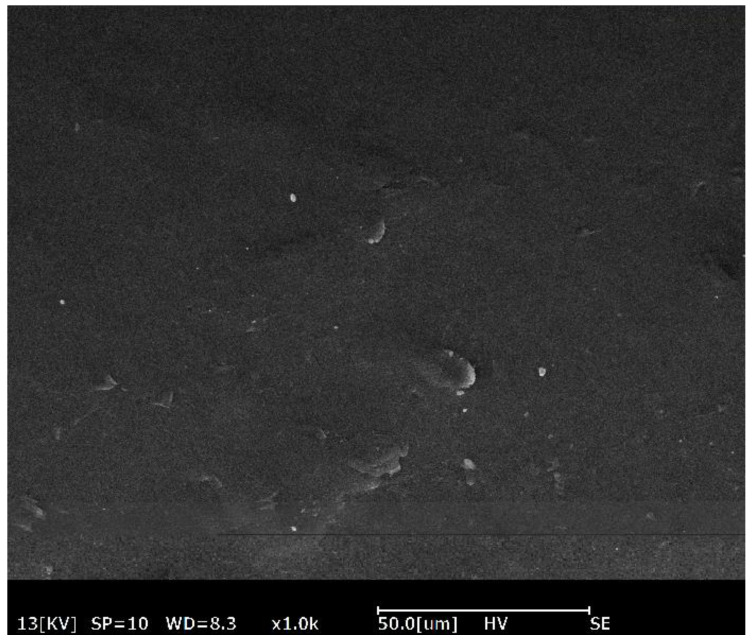
The SEM images of the 3D printed composite material samples.

MWCNTs-COOH was mixed with PA6 and POE-g-MAH in specific proportions to prepare a masterbatch with an MWCNTs content of 15 wt.%. The high-filler-content masterbatch was then blended with PA6 and POE-g-MAH to fabricate PA6/POE-g-MAH/MWCNTs composite strands with MWCNTs contents of 2.5, 5, 7.5, 10, 12.5, and 15 wt. Similarly, BN was combined with PA6 and POE-g-MAH in certain proportions to prepare a PA6/POE-g-MAH/BN masterbatch with a BN content of 20 wt.%, which was subsequently used to produce composite strands with BN contents of 5 wt.% and 10 wt.%. The specific mass fractions of the different components are listed in Table 1. Regarding the scanning electron microscopy (SEM) analysis of the surface morphology of the polymer melt blend, the image depicted in Figure 3 reveals the microstructural characteristics of the polymer matrix. At a magnification of 1000x, the surface of the polymer matrix exhibits significant uniformity, with no apparent undulations or cracks observed. This observation strongly confirms the high smoothness of the polymer matrix surface and also indicates that the filler has achieved a uniform distribution within the polymer matrix.

### 2.4. Gradient Composite Materials Prepared by S2-FDM 3D Printing

The PA6/POE-g-MAH, PA6/POE-g-MAH/MWCNTs, and PA6/POE-g-MAH/BN composites with varying filler ratios were dried at 80 °C for 12 h in an oven. Subsequently, the gradient composites were printed using a Morioka M2030X FDM 3D printer. It is important to load the left inlet with PA6/POE-g-MAH/MWCNTs or PA6/POE-g-MAH/BN filaments, whereas the right inlet should be loaded with pure PA6/POE-g-MAH filaments to ensure that the nozzles are not clogged during the printing process. Cura 21.01.28 software must be used to set precise print parameters, including gradient and color mixing modes. After pre-experimental verification, the optimal combination of printing parameters was determined to be a nozzle temperature of 240 °C, hot-bed temperature of 30 °C, and print speed of 40 mm/s. The specific printer parameters are listed in Table 2. To differentiate between the materials, the naming convention of A → B is used to label the gradient materials, indicating that the material gradually transitions from A to B from the bottom to the top.

### 2.5. Homogeneous Materials Were Prepared by Single-Feed FDM 3D Printing

The homogeneous materials were successfully prepared as the reference samples using a standard single-input, single-output FDM 3D printing process. During the fabrication, constant filler content was maintained for each layer. After rigorous mathematical calculations, we concluded that the filler contents of the gradient materials A–B were equivalent to that of the (B/2) homogeneous material.

### 2.6. Testing and Characterization

In this study, we employed a differential scanning calorimeter (DSC-Q200, TA Instruments) to precisely measure the melting behavior of the blends. The experiments were conducted under strictly controlled conditions at a constant heating rate of 10 °C/min to ensure consistency in the tests. The testing temperature range was set from 30 to 250 °C, covering the melting and crystallization temperature ranges of the materials. The degree of crystallinity of the blends was calculated using the following formula:

(1) $$\chi_{c}=\frac{\Delta H_{m}}{\omega \times \Delta H_{m}^{\prime }}$$

where ΔH_m_ is the enthalpy of melting of the blend, ω is the mass fraction of PA6 in the blend, and ΔH’_m_ is the enthalpy of melting of the polymer when it reaches 100% crystallinity, which was chosen to be ΔH’_m_ = 190 J/g for PA6 according to Ref. [27]. To verify the accuracy of the measurements, we conducted three independent DSC tests for each sample and calculated their mean values and standard deviations.

The warpage of the material was measured by printing a rectangular sample with dimensions 40 mm × 10 mm × 3 mm and the warpage was calculated using the following equation:

(2) $$w=\frac{H_{1}-H_{2}}{H_{1}}\times 100\%$$

**Figure 4:**
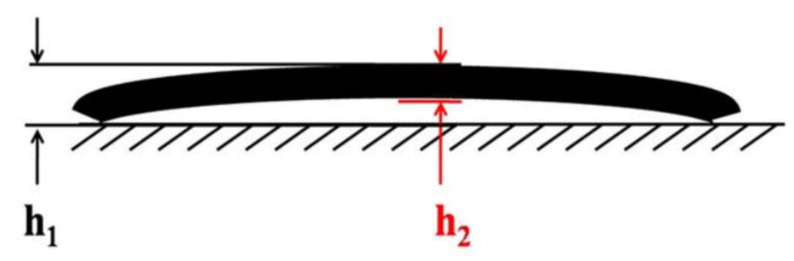
The schematic diagram of the testing of the warpage degree.

The specimen was inverted and placed on the table, with H_1_ representing the distance between the highest point of the specimen and the plane and H_2_ indicating the thickness of the specimen, as shown in Figure 4. To minimize human error, all the measurements were conducted by the same operator using identical instruments. The warpage of each specimen was measured five times, and the average value was taken as the final result.

In addition, to estimate the crystal size of the nanocomposites, we used the Scherrer equation, an analytical method based on the X-ray diffraction (XRD) peak width. The crystal size can be calculated by the following formula:

(3) $$D=\frac{K\lambda }{\beta COS(\theta )}$$

where K is the Scherrer constant, λ is the wavelength of the X-ray, β is the half-height width (FWHM) of the diffraction peak, and θ is the Bragg Angle. We performed at least three XRD scans for each sample and calculated the FWHM of the diffraction peak. The crystal sizes were calculated based on the average of these measurements. Using these methods, we ensured the accuracy and reliability of the experimental results. All the tests were conducted in a controlled environment to reduce the impact of environmental factors on the experimental results.

The X-ray diffraction scans of the materials were performed using an X-ray diffractometer (Miniflex 600, Rigaku, Japan) with a Cu-target Kα radiation source in the range of 10°–40°. The mechanical properties of the materials were evaluated using a universal testing machine (AGS-X PLUS; Shimadzu Corporation, Kyoto, Japan). The tensile tests were conducted on dumbbell-shaped specimens measuring 75 mm × 12.5 mm × 2 mm, with a tensile rate of 5 mm/min, and the slope at a strain range of 0.05%–0.25% was taken as the tensile modulus of the specimen. The flexural tests were performed on rectangular specimens measuring 70 mm × 10 mm × 3.5 mm, with the indenter moving at a speed of 2 mm/min, and the slope at a strain range of 0.05%–0.25% was taken as the flexural modulus of the specimen. The interlaminar shear tests were conducted on rectangular specimens measuring 20 mm × 10 mm × 2 mm, with the indenter loading at a rate of 2 mm/min.

## 3. Results and Discussions

### 3.1. Preparation and Characterization of PA6/POE-g-MAH

**Figure 5:**
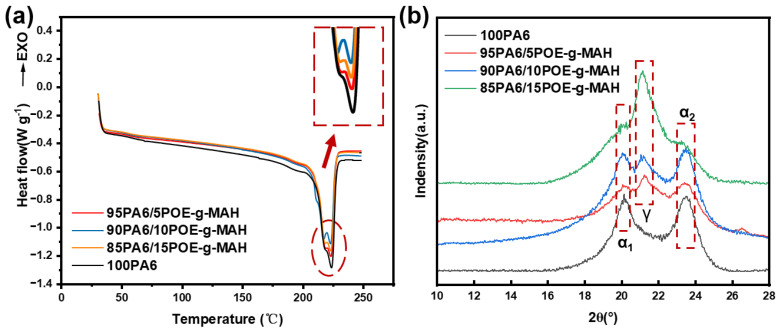
(**a**) DSC curves; (**b**) XRD spectra of PA6 and PA6/POE-g-MAH blends.

As a semi-crystalline polymer, the internal molecular chain arrangement of pure PA6 significantly affects the warpage of the final part. As depicted in Figure 5a, the secondary melting curves of the PA6 and PA6/POE-g-MAH blends revealed that PA6 exhibited a clear melting peak at 224 °C, which primarily corresponded to the melting of the α-crystalline type. However, the addition of POE-g-MAH resulted in the melting curves of the blends displaying distinct melting peaks near 218 and 224 °C, corresponding to the γ-crystalline and α-crystalline types, respectively [28,29,30]. As the concentration of POE-g-MAH was increased, the melting peak associated with the crystalline type became more pronounced. This can be attributed to the effect of POE-g-MAH on the movement of the PA6 molecular chains, making it harder for them to fit into the crystal lattice and reduce their regularity. Consequently, some of the crystal structures were incomplete, which led to the appearance of a double melting peak. As shown in Table 3, the crystallinity of the material was reduced after incorporating POE-g-MAH, which further established that the molecular chain arrangement of the blends tended to be irregular. Figure 5b shows the X-ray diffraction (XRD) plots of the PA6/POE-g-MAH composites with various mass ratios. Pure PA6 displayed two diffraction peaks near 2θ = 20.2° and 23.5°, which correspond to the α1-crystalline phase (200) crystallographic diffraction and α2-crystalline phase (002, 220) crystallographic diffraction of the PA6 molecular chain, respectively. However, upon addition of POE-g-MAH, a diffraction peak appeared in the blend near 2θ = 21.6°, primarily corresponding to the γ-crystalline phase (200, 001) crystal surface diffraction peaks. Scherrer’s equation estimated the crystal size to be 6.90 nm. With an increase in the POE-g-MAH content, the α-peak of the blends progressively increased, whereas the γ-peak progressively weakened. Typically, the α-crystalline phase is the thermodynamically stable phase of PA6, comprising hydrogen-bonded chain sheets formed between antiparallel chains, whereas the γ-crystalline phase is a metastable phase, comprising random hydrogen bonds between parallel chains [31,32,33]. Therefore, the main reason for the peak in the heat absorption of the PA6/POE-g-MAH blends was the decomposition of hydrogen bonding. Both the DSC and XRD results showed that POE-g-MAH, which has a large number of short-chain branches, disrupted the regular arrangement of the PA6 molecular chains and caused the formation of an unstable γ-crystalline phase. This irregular arrangement of molecular chains leads to less shrinkage of the printed part. However, it is important to note that adding polyolefin elastomers reduces the strength of PA6 [23,34,35]. Therefore, the addition of POE-g-MAH should be minimized while ensuring a controllable wire preparation. Based on this principle, the matrix for this experiment was chosen as 95PA6/5POE-g-MAH, and a gradient structure was constructed on this basis.

### 3.2. Preparation of Gradient Structure and Warpage Test

**Figure 6:**
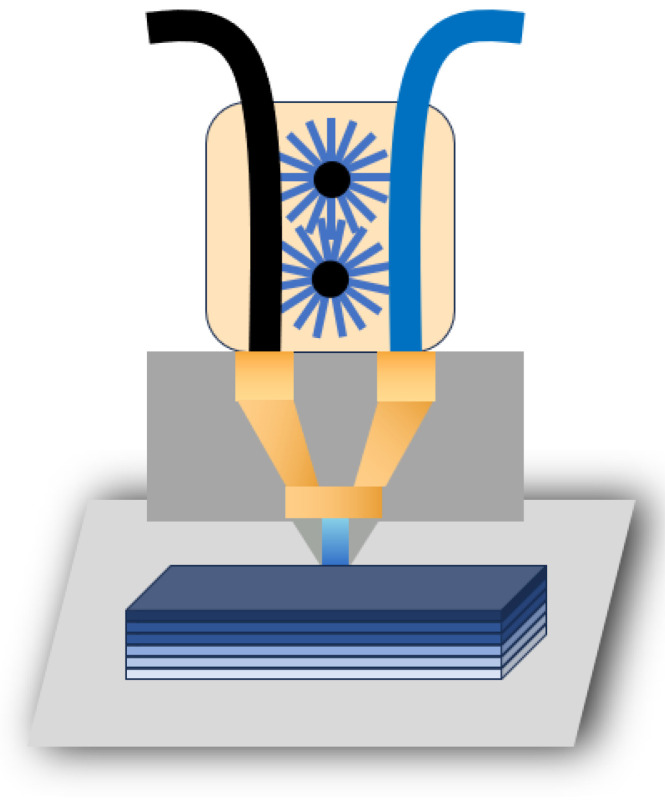
The schematic of the PA6-based gradient material prepared using S2-FDM.

The diagram in Figure 6 shows the method of generating PA6-based gradient materials using single-nozzle two-component composite fused deposition molding (S2-FDM). This process employs carboxylated multi-walled carbon nanotubes (COOH-MWCNTs) and boron nitride (BN) as dimensional stability modifiers for PA6, resulting in 3D-printable PA6 composite filaments with a high filler content. The left portion of the diagram shows a filament with a substantial amount of filler, whereas the right portion shows a pure PA6/POE-g-MAH filament. The entry of the filament into the motor can be precisely controlled, allowing for the efficient combination of the two materials in varying ratios and printing of layers with increasing filler content.

**Figure 7:**
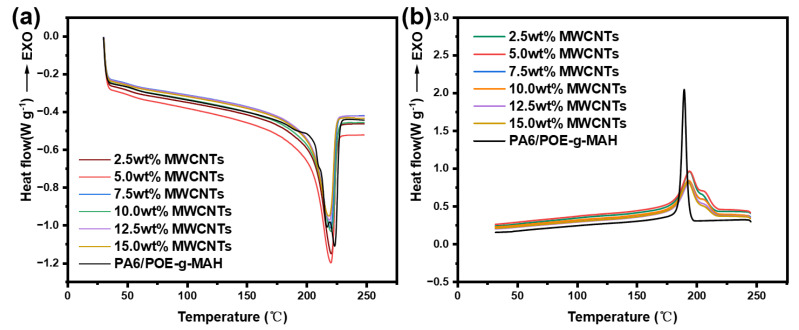
DSC curves of PA6/POE-g-MAH/MWCNTs composites: (**a**) secondary heating curve and (**b**) cooling curve.

Figure 7 depicts the second heating and cooling curves of the PA6/POE-g-MAH/MWCNTs obtained by differential scanning calorimetry (DSC). From the heating curve in Figure 7a, it is evident that PA6/POE-g-MAH exhibits two distinct melting peaks at approximately 218 and 224 °C. Upon the incorporation of MWCNTs, the melting curve of the composite material exhibited a single melting peak. From Table 4, it can be observed that the crystallinity of the composite material decreased with increasing MWCNTs content. This decrease in crystallinity may be attributed to the hindrance of the PA6 molecular chain mobility by MWCNTs, which restricts the growth of polymeric crystals, leading to a reduction in the crystallinity of the PA6/POE-g-MAH/MWCNTs composite. The cooling curve in Figure 7b demonstrates that the introduction of MWCNTs resulted in a dual-peak crystallization pattern in the composite material. Both the initial crystallization temperature and overall crystallization temperature were elevated, and the peak shape broadened. This indicates that MWCNTs, acting as heterogeneous nucleating agents, can reduce the degree of supercooling required for polymer crystallization, enhancing the crystallization rate, and consequently lead to an increase in the crystallization temperature. The emergence of crystallization double peaks may be the result of two contributing factors. Firstly, the presence of MWCNTs limits the thermal motion of PA6 molecular chains, restricting the melting of PA6 crystals upon heating and promoting the perfection of PA6 crystallization through heterogeneous nucleation at higher MWCNTs content during cooling. Secondly, due to the nanotube structure of MWCNTs, there are interstitial gaps between closely packed MWCNTs, where PA6 chain segments may not fully crystallize, resulting in the appearance of crystallization double peaks upon cooling.

**Figure 8:**
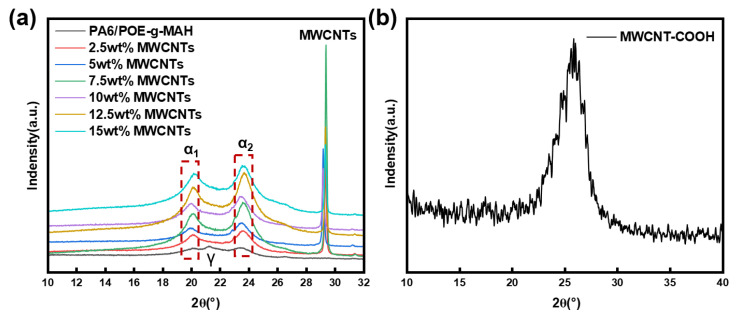
XRD spectra of (**a**) PA6/POE-g-MAH/MWCNTs composites and (**b**) MWCNT-COOH.

XRD tests were conducted to thoroughly examine the crystalline properties of the PA6/POE-g-MAH/MWCNTs composites. As depicted in Figure 8, the XRD spectra of the different composites exhibited the characteristic diffraction peaks of the MWCNTs after the addition of the fillers. By comparing the XRD peaks of the MWCNT-COOH powders, as shown in Figure 8b, we observed a right shift in the diffraction peaks of the MWCNTs. This increase in angle, according to Bragg’s equation, implies a decrease in the crystallite spacing. This may be attributed to the interaction between PA6 and the MWCNTs, which resulted in the formation of defects in the MWCNTs, thus causing their cell parameters to decrease. Furthermore, the diffraction peaks close to 20.2° and 23.5° corresponded to the α1 crystallographic phase (200) crystal plane and the α2 crystallographic phase (002, 220) crystal planes of the PA6 molecular chain, respectively. In contrast, PA6/POE-g-MAH exhibited a diffraction peak near 21.3°, which corresponded to the γ-crystalline phase (200,001) crystal plane. However, with the addition of multi-walled carbon nanotubes (MWCNTs), the γ-crystalline phase disappeared and the diffraction peak at the α2 crystal plane tended to shift to the right. Notably, as the MWCNT content increased, the diffraction peaks of the composites became higher and sharper, indicating that the degree of ordering and crystallinity of the PA6 molecular chains were enhanced. This may be attributed to the fact that the MWCNTs acted as heterogeneous nucleation sites and promoted the further crystallization of PA6. Consequently, only the thermodynamically stable α-crystalline phase appeared in the PA6/POE-g-MAH/MWCNTs composites. The X-ray diffraction (XRD) test result aligns with previous differential scanning calorimetry (DSC) results, further validating our conclusions.

**Figure 9:**
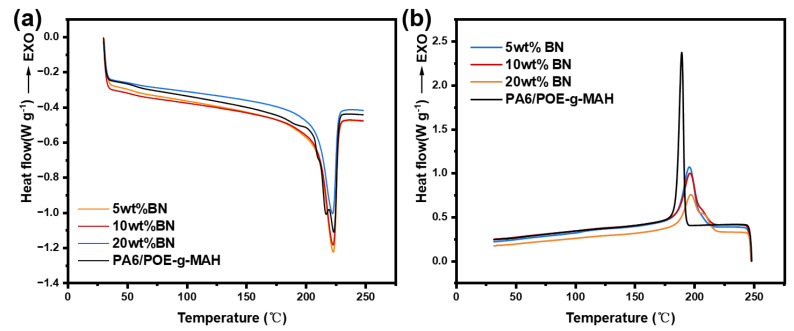
DSC curves of PA6/POE-g-MAH/BN composites: (**a**) secondary heating curve and (**b**) cooling curve.

The DSC secondary heating and cooling curves for PA6/POE-g-MAH/BN are shown in Figure 9. The melting peaks for PA6/POE-g-MAH at 216.9 °C and 223.3 °C correspond to α- and γ-crystalline melting, respectively. Upon the addition of BN, the γ-crystalline melting peaks disappeared, which aligns with the trend observed in the DSC heating curves of the PA6/POE-g-MAH/MWCNTs. As shown in Table 5, the crystallinity of the composites decreases with increasing BN content. This is attributed to the fact that BN hinders the regular arrangement of PA6 molecular chains, making it difficult to form large crystals in the composites and thereby leading to a decrease in crystallinity. From the cooling curves in Figure 9b, it can be observed that the crystallization peaks of PA6/POE-g-MAH were sharper. As the BN content increased, the onset of crystallization and crystallization peak temperatures of the composites increased, the bimodal crystallization peaks became more pronounced, and the peak shape widened. This may be because BN acts as a nonhomogeneous nucleating agent in the matrix, which enhances the crystallization rate [36].

**Figure 10:**
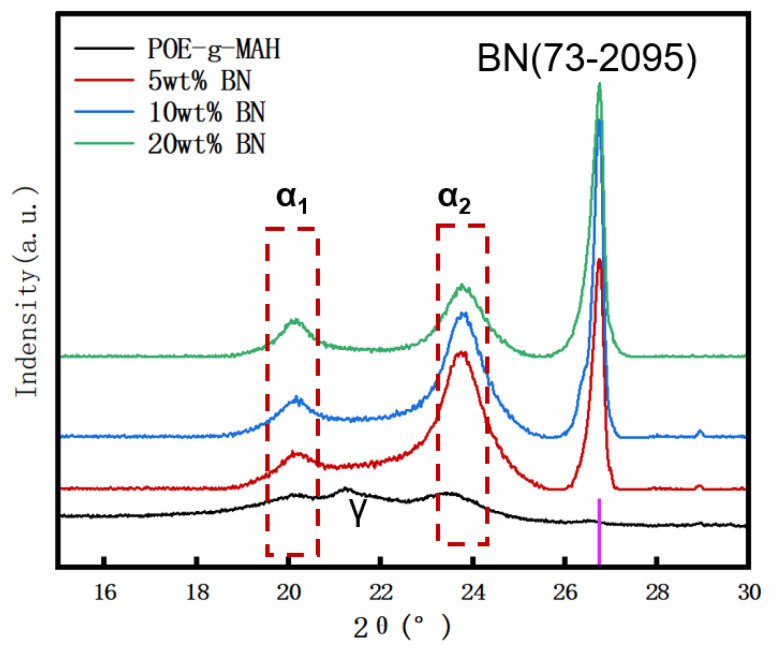
XRD spectra of PA6/POE-g-MAH/MWCNTs composites.

Figure 10 shows the XRD spectrum of the PA6/POE-g-MAH/BN. In this graph, it can be observed that the incorporation of BN resulted in the addition of the characteristic diffraction peaks of h-BN to the XRD curves of the composites. Scherrer’s equation estimated the crystal size to be 25.21 nm. Specifically, the diffraction peaks located near 20.1° and 23.7° corresponded to the α1 crystallographic phase (200) crystal plane and the α2 crystallographic phase (002, 220) crystal plane of the PA6 molecular chain, respectively. Furthermore, compared to the XRD spectra of the PA6/POE-g-MAH/MWCNTs, the introduction of BN led to the disappearance of the γ-crystalline phase diffraction peaks, whereas the α1- and α2-crystalline phase diffraction peaks became more prominent. This suggests that BN promotes the formation of thermodynamically stable α-crystalline forms, which is consistent with the results of DSC analysis.

Given its notable crystallinity, PA6 is prone to issues such as bending, warping, and interlayer peeling during the printing process, which may result in incomplete printing. To enhance the dimensional stability of the material, we optimized the printing parameters, including the wall thickness and adhesion. The experimental results indicated that by setting the wall thickness to 0.8 mm and increasing the number of attachments to 10, the degree of warpage in the printed parts could be significantly improved without affecting the material properties. Building on this, we prepared nonhomogeneous materials, PA6/POE-g-MAH/MWCNTs and PA6/POE-g-MAH/BN, with different gradient ratios. We also determined the degree of warpage of these materials when the rectangular print samples were inverted. For convenience, in future studies, we will assign sample numbers to each sample, as listed in Table 6.

**Figure 11:**
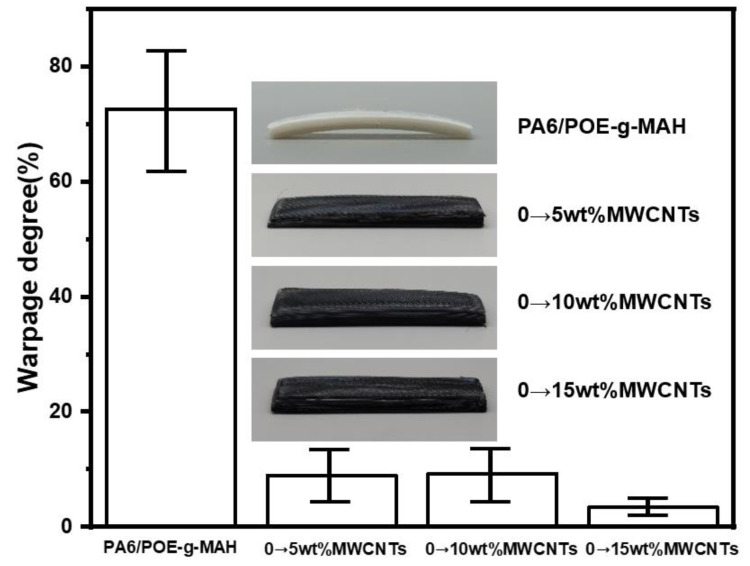
Warpage of PA6/POE-g-MAH/MWCNTs gradient materials.

Following extensive observation and analysis of the PA6 3D-printed samples, we discovered that there are critical issues with warping, deformation, and a lack of dimensional stability, which significantly hinder the material’s performance and potential for use. Figure 11 shows the warping data for the PA6/POE-g-MAH/MWCNTs gradient materials. As shown in the figures, PA6/POE-g-MAH exhibits a remarkable degree of bending with a warping level as high as 72.64%. However, these materials are not suitable for practical applications. However, by incorporating a gradient content structure of MWCNTs, we successfully reduced the warping of PA6 composites. The experimental results revealed that the samples had flat surfaces without warping. Furthermore, warping reached its lowest value of 3.40% under a gradient content of 0–15 wt.% MWCNTs, which provides strong support for further optimization of the material properties.

**Figure 12:**
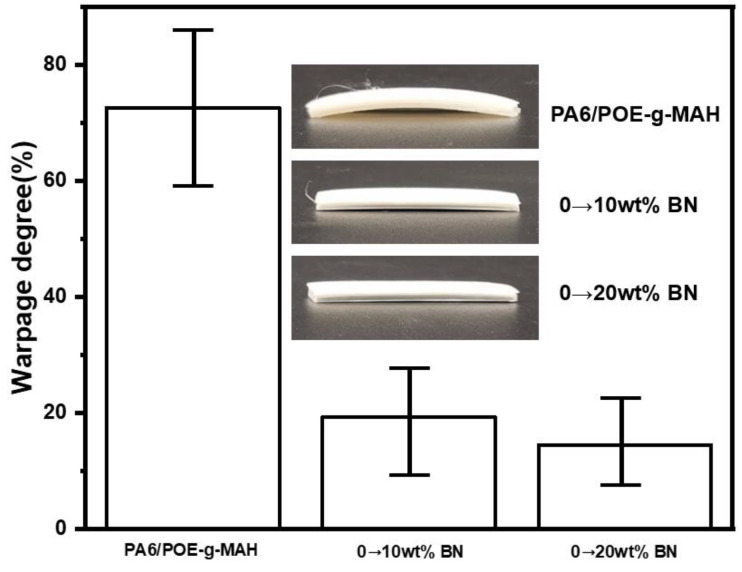
Warpage of PA6/POE-g-MAH/BN gradient materials.

The variation in warpage of the PA6/POE-g-MAH/BN gradient materials is shown in Figure 12. It is apparent from the figures that the incorporation of the BN gradient structure significantly reduces the warpage of the printed part. As the gradient content increased, the warpage progressively declined. When the BN content increased from 0 to 20 wt.%, the warpage decreased to 14.51%. The experimental data revealed that both the MWCNTs and BN gradient structures could effectively address the bending and warping problems of PA6 prints owing to shrinkage internal stresses. This results in a significant improvement in the dimensional stability of the composites. This is because, during the printing process, the temperature of the upper layer (layer N + 1) is higher than that of the lower layer (layer N), making the molecular chain movement of the layer (N + 1) more active and the volume shrinkage greater. By designing the filler content to increase layer by layer, the shrinkage of each layer of the printed part shows a decreasing trend, which helps offset the shrinkage internal stress between adjacent stacked layers during the FDM printing process, thus enhancing the dimensional stability of PA6 during the 3D printing process.

### 3.3. Mechanical Properties

**Figure 13:**
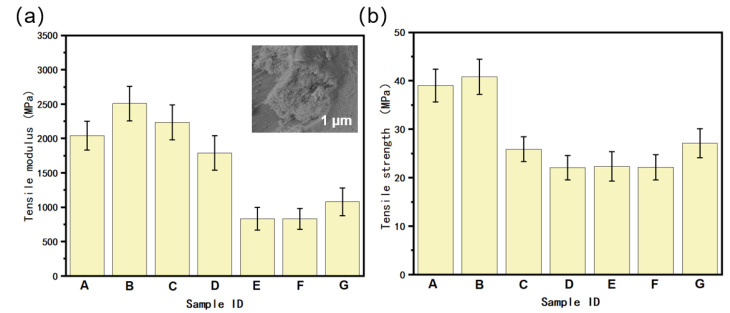
The tensile properties of PA6/POE-g-MAH/MWCNTs composite materials: (**a**) tensile modulus (inset shows the scanning electron microscopy image of sample D), (**b**) tensile strength. (A: 95PA6/5POE-g-MAH. B: PA6/POE-g-MAH/MWCNTs-COOH (2.5 wt.%). C: PA6/POE-g-MAH/MWCNTs-COOH (5 wt.%). D: PA6/POE-g-MAH/MWCNTs-COOH (7.5 wt.%). E: PA6/POE-g-MAH → POE-g-MAH/MWCNTs-COOH (5 wt.%). F: PA6/POE-g-MAH → POE-g-MAH/MWCNTs-COOH (10 wt.%). G: PA6/POE-g-MAH → POE-g-MAH/MWCNTs-COOH (15 wt.%)).

The relationship between the gradient structure and mechanical properties was studied, revealing that, unlike traditional homogeneous materials, gradient composites exhibit anisotropy in their mechanical properties. To investigate this relationship more precisely, systematic mechanical property tests were conducted on various homogeneous and gradient samples with different filler contents. In the tensile tests, the loading direction was perpendicular to the deposition direction to ensure that the testing was not influenced by the loading direction. The results of the tensile tests of the PA6/POE-g-MAH/MWCNTs composites are shown in Figure 13. These results indicate that the incorporation of MWCNTs into homogeneous composites can effectively enhance the tensile strength and modulus of the materials. The tensile strength of the composites peaked at an MWCNT content of 2.5 wt.%. The mechanical properties of the composites, specifically their tensile strength and modulus, decreased with the content. This can be observed in the inset of Figure 13a, where a higher MWCNT content is more likely to agglomerate, leading to difficulty in uniformly dispersing fillers within the material. When a material is subjected to external forces, the agglomerated fillers act as mechanical defect points, resulting in a reduction in the mechanical properties of the composites.

Unlike homogeneous composites, gradient composites exhibit dissimilar trends. As the gradient content of MWCNTs increased, the tensile modulus and strength of the materials increased consistently. However, the tensile properties of the PA6/POE-g-MAH/MWCNTs gradient composites were not as good as those of pure PA6/POE-g-AH and homogeneous composites. This may be due to the fact that in gradient composites, the content of top filler is twice as much as in homogeneous composites, as the large introduction of MWCNTs leads to severe agglomeration phenomena, which in turn affects their mechanical properties. After calculation, we found that the content of MWCNTs in the half-thickness samples of the gradient composites was higher than that in the homogeneous materials, which may be the primary reason for the inferior tensile properties of this part compared with those of homogeneous materials. According to the crack-diffusion mechanism, when the samples were subjected to external forces, the cracks tended to diffuse along the weakest region, which was one of the reasons for the inferior tensile properties of the PA6/POE-g-MAH/MWCNTs gradient composites. Nevertheless, when the gradient content of MWCNTs was increased from 0 to 15 wt.%, the tensile strength of the composites reached 27.3 MPa, which was superior to that of the PA6/polyolefin elastomer composites fabricated by FDM 3D printing [23,24].

**Figure 14:**
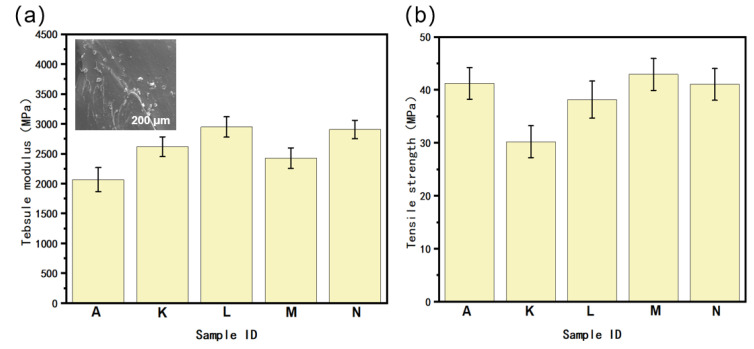
The tensile properties of PA6/POE-g-MAH/BN composite materials: (**a**) tensile modulus (with an inset showing the scanning electron microscopy image of sample L), (**b**) tensile strength. (A: 95PA6/5POE-g-MAH. K: PA6/POE-g-MAH/BN (5 wt.%). L: PA6/POE-g-MAH/BN (10 wt.%). M: PA6/POE-g-MAH → POE-g-MAH/BN (10 wt.%). N: PA6/POE-g-MAH → POE-g-MAH/BN (20 wt.%)).

The tensile properties of the PA6/POE-g-MAH/BN composites are shown in Figure 14. This study revealed that incorporating BN led to a noticeable improvement in the tensile modulus of the PA6/POE-g-MAH homogeneous composites while causing a slight decrease in tensile strength. Additionally, it was observed that the addition of the BN gradient structure to nonhomogeneous materials resulted in a significant enhancement of the tensile modulus, with an increase of up to 43.08% when the BN gradient content was increased from 0 to 20 wt.%. However, it is important to note that this gradient structure did not have a significant impact on the tensile strength of the composites, which was consistent with the matrix material. Even in sample N, which had a relatively poor tensile strength performance, the tensile strength reaches 41.04 MPa. This suggests that the introduction of the BN gradient structure maintains the dimensional stability of the PA6-based composites without compromising their mechanical properties. The BN gradient materials exhibited superior tensile properties compared with the PA6/POE-g-MAH/MWCNTs gradient composites. Additionally, as shown in the inset of Figure 14a, there was relatively less agglomeration of BN, creating fewer mechanical defects in the composite. This micron-sized flake filler was easily oriented along the print plane after extrusion through the nozzle parallel to the loading direction of the tensile test. Thus, BN can be used as a mechanical reinforcement filler to optimize the tensile properties of the composites.

**Figure 15:**
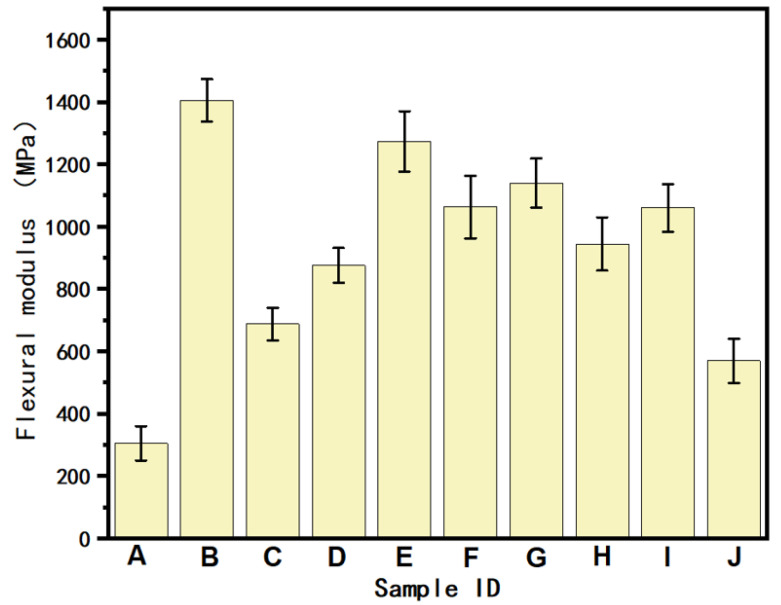
Flexural modulus of PA6/POE-g-MAH/MWCNTs composite. (A: 95PA6/5POE-g-MAH. B: PA6/POE-g-MAH/MWCNTs-COOH (2.5 wt.%). C: PA6/POE-g-MAH/MWCNTs-COOH (5 wt.%). D: PA6/POE-g-MAH/MWCNTs-COOH (7.5 wt.%). E: PA6/POE-g-MAH → POE-g-MAH/MWCNTs-COOH (5 wt.%). F: PA6/POE-g-MAH → POE-g-MAH/MWCNTs-COOH (10 wt.%). G: PA6/POE-g-MAH → POE-g-MAH/MWCNTs-COOH (15 wt.%). H: POE-g-MAH/MWCNTs-COOH (5 wt.%) → PA6/POE-g-MAH. I: POE-g-MAH/MWCNTs-COOH (10 wt.%) → PA6/POE-g-MAH. J: POE-g-MAH/MWCNTs-COOH (15 wt.%) → PA6/POE-g-MAH).

Figure 15 shows the flexural moduli of the PA6/POE-MAH-MWCNTs composites. The addition of MWCNTs significantly improved the flexural modulus of the composites, mainly because of the high stiffness of the MWCNTs themselves, which was reinforced by a mechanism similar to that of conventional reinforcement materials. However, the flexural modulus showed a decreasing trend as the MWCNTs content further increased. For the gradient materials, when the MWCNTs side was in contact with the indenter (i.e., samples E, F, and G, and the force loading direction was from the PA6/POE-g-MAH matrix composite to PA6/POE-g-MAH), the flexural modulus was higher than that of the pure PA6/POE-g-MAH side in contact with the indenter (i.e., samples H, I, and J, and the force loading direction was from PA6/POE-g-MAH to the PA6/POE-g-MAH matrix composite). This may be because the introduction of MWCNTs enhances the flexural strength of the material, which enables the PA6/POE-g-MAH/MWCNTs side to withstand greater stresses. Additionally, the flexural moduli of the nonhomogeneous samples E and F exceeded those of the homogeneous printed samples C and D when the gradient content of MWCNTs was 0–10 wt.% and 0 → 15 wt.%, respectively.

**Figure 16:**
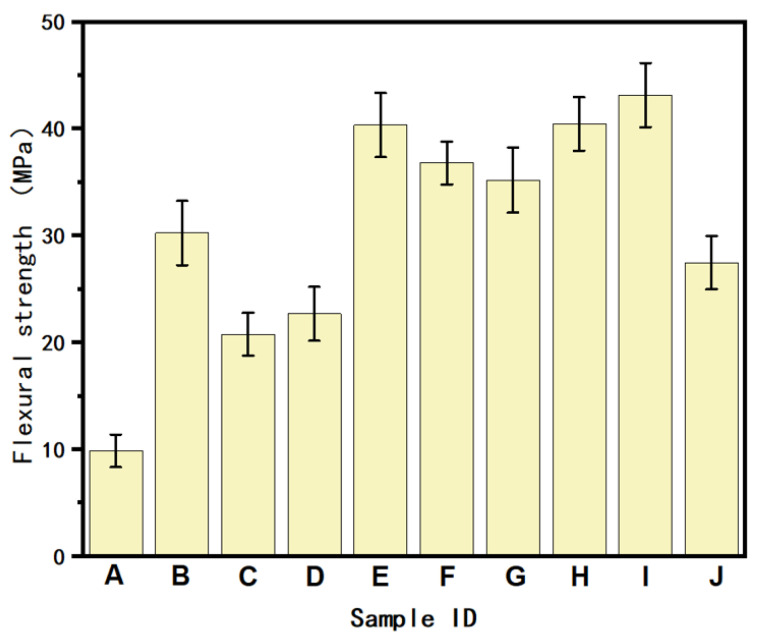
Flexural strength of the PA6/POE-g-MAH/MWCNTs composite. (A: 95PA6/5POE-g-MAH. B: PA6/POE-g-MAH/MWCNTs-COOH (2.5 wt.%). C: PA6/POE-g-MAH/MWCNTs-COOH (5 wt.%). D: PA6/POE-g-MAH/MWCNTs-COOH (7.5 wt.%). E: PA6/POE-g-MAH → POE-g-MAH/MWCNTs-COOH (5 wt.%). F: PA6/POE-g-MAH → POE-g-MAH/MWCNTs-COOH (10 wt.%). G: PA6/POE-g-MAH → POE-g-MAH/MWCNTs-COOH (15 wt.%). H: POE-g-MAH/MWCNTs-COOH (5 wt.%) → PA6/POE-g-MAH. I: POE-g-MAH/MWCNTs-COOH (10 wt.%) → PA6/POE-g-MAH. J: POE-g-MAH/MWCNTs-COOH (15 wt.%) → PA6/POE-g-MAH).

Figure 16 depicts the flexural strength of the PA6/POE-g-MAH/MWCNTs composites. Although the FDM 3D printing process causes warping and delamination in the PA6 material, resulting in reduced interlayer bonding properties and a lower flexural strength of 9.51 MPa, the gradient material still outperforms homogeneous materials with equal material contents. When the gradient content is 0–10 wt.%, the flexural modulus can reach 43.14 MPa if the force is applied from the pure polymer side to the PA6/POE-g-MAH/MWCNTs side, representing a 354% improvement over the pure polymer and a 108% improvement over the homogeneous printed samples with 5 wt.% MWCNTs. These findings confirm that the gradient structure of MWCNTs can effectively optimize the flexural strength of PA6 3D-printed composites.

**Figure 17:**
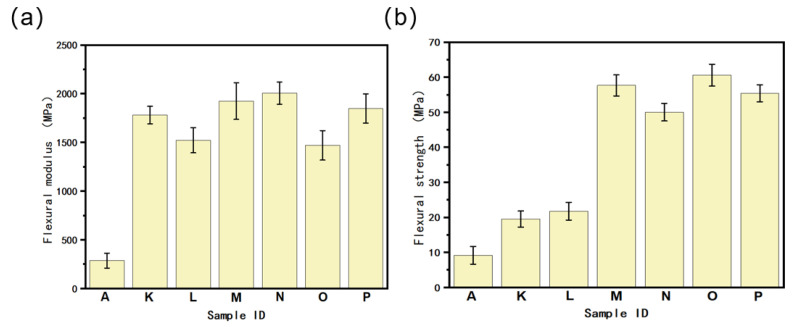
(**a**) Flexural modulus and (**b**) flexural strength of PA6/POE-g-MAH/BN composites. (A: 95PA6/5POE-g-MAH. K: PA6/POE-g-MAH/BN (5 wt.%). L: PA6/POE-g-MAH/BN (10 wt.%). M: PA6/POE-g-MAH → POE-g-MAH/BN (10 wt.%). N: PA6/POE-g-MAH → POE-g-MAH/BN (20 wt.%). O: POE-g-MAH/BN (10 wt.%) → PA6/POE-g-MAH. P: POE-g-MAH/BN (20 wt.%) → PA6/POE-g-MAH).

Figure 17 shows the flexural performance of the PA6/POE-g-MAH/BN composite. As shown in the figure, the incorporation of BN fillers significantly boosts the flexural properties of both homogeneous and nonhomogeneous materials, which mirrors the MWCNTs composite system. Notably, for the same PA6/POE-g-MAH/BN gradient material, there was a substantial difference in the flexural modulus when loaded from different directions. An additional analysis revealed that the bending strengths of the gradient materials exceeded those of the homogeneously printed materials with corresponding BN contents. In particular, when the BN content gradient was 0–10 wt.% and the loading direction was from the pure polymer side to the PA6/POE-g-MAH/BN side (Sample O), the flexural strength of the composites reached an impressive 60.91 MPa, which was a remarkable improvement of 541% compared to the pure polymer and 207% compared to the 5 wt.% BN-homogenized printed sample. In conclusion, the introduction of MWCNTs and BN gradient structures can effectively enhance the bending properties of PA6, sometimes surpassing those of homogeneous 3D printing materials. Furthermore, the same material exhibits different bending properties under various loading directions, providing the potential for “on-demand supply”. This allows for a simple flip-flop operation to satisfy the material’s mechanical property requirements, thus significantly reducing materials and costs.

**Figure 18:**
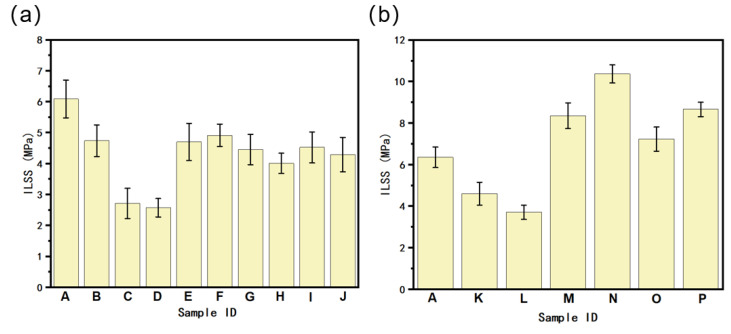
Interlaminar shear strengths of PA6 composites (**a**): PA6/POE-g-MAH/MWCNTs composite; (**b**): PA6/POE-g-MAH/BN composite). (A: 95PA6/5POE-g-MAH. B: PA6/POE-g-MAH/MWCNTs-COOH (2.5 wt.%). C: PA6/POE-g-MAH/MWCNTs-COOH (5 wt.%). D: PA6/POE-g-MAH/MWCNTs-COOH (7.5 wt.%). E: PA6/POE-g-MAH → POE-g-MAH/MWCNTs-COOH (5 wt.%). F: PA6/POE-g-MAH → POE-g-MAH/MWCNTs-COOH (10 wt.%). G: PA6/POE-g-MAH → POE-g-MAH/MWCNTs-COOH (15 wt.%). H: POE-g-MAH/MWCNTs-COOH (5 wt.%) → PA6/POE-g-MAH. I: POE-g-MAH/MWCNTs-COOH (10 wt.%) → PA6/POE-g-MAH. J: POE-g-MAH/MWCNTs-COOH (15 wt.%) → PA6/POE-g-MAH. K: PA6/POE-g-MAH/BN (5 wt.%). L: PA6/POE-g-MAH/BN (10 wt.%). M: PA6/POE-g-MAH → POE-g-MAH/BN (10 wt.%). N: PA6/POE-g-MAH → POE-g-MAH/BN (20 wt.%). O: POE-g-MAH/BN (10 wt.%) → PA6/POE-g-MAH. P: POE-g-MAH/BN (20 wt.%) → PA6/POE-g-MAH).

The weakest point in the FDM 3D-printed samples is the interface between two adjacent layers; therefore, we conducted tests on the interlaminar shear strength (ILSS) of the composites. The results depicted in Figure 18 indicate a declining trend in the interlaminar shear strength of the homogeneously printed samples after the addition of the two fillers. However, when the high-filler-content side was in contact with the indenter (i.e., samples E, F, G, M, and N, with the force loading direction from the high-filler-content side to the pure polymer side), the samples displayed better interlaminar shear strengths than those in which the pure polymer side was in contact with the indenter (i.e., H, I, J, O, and P). This could be attributed to the fact that stresses are transferred downward when the high-filler-content side is under stress, whereas the higher matrix content at the bottom, combined with the fact that the pure polymer exhibits the highest ILSS value compared to the homogeneous composites, effectively increases the interlaminar shear strength of the composites. In addition, the interlayer shear strength of the gradient materials was consistently higher than that of homogeneous materials containing the same amount of filler. This can be attributed to the decrease in the melt viscosity of the extruded wires at higher filler contents, which leads to weaker wire-to-wire and layer-to-layer bonding. In contrast, the gradient structure is formed by gradually mixing pure polymer wires and high-filler wires layer by layer. This results in increased molecular entanglement and diffusion between layers and stronger bonding, leading to greater interlayer shear strength than homogeneous materials with high filler content. Notably, the PA6/POE-g-MAH/BN gradient material exhibited exceptional interlaminar strength. When the BN content gradient was 0–20 wt.% and the force loading direction was from the PA6/POE-g-MAH/BN side to the polymer side (i.e., sample N), the interlaminar shear strength of the samples reached 10.23 MPa, which was a 64% improvement compared to the substrate and 173% compared to the 10 wt.% BN homogeneous printed sample. This further confirms the advantages of the gradient structure in enhancing the interlayer shear strength of the FDM 3D-printed samples.

## 4. Conclusions

In this experiment, we successfully prepared gradient-structured PA6 composites using single-nozzle two-component composite fused deposition molding (S2-FDM) printing technology. MWCNTs and BN were used as dimensional stabilizers, and we precisely regulated the mixing ratios of PA6/POE-g-MAH/MWCNTs, PA6/POE-g-MAH/BN wires, and PA6/POE-g-MAH, which were varied using the dual feeding system of the printing equipment to create a gradient structure. The results of the DSC and XRD analyses indicated that the incorporation of fillers led to the disappearance of the unstable γ-crystalline form and increased the thermodynamically stable α-crystalline form. The warpage test results demonstrated that the gradient structure effectively improved the warpage problem in the PA6 3D-printed parts and significantly enhanced the dimensional stability of the material. The tensile testing demonstrated that the PA6/POE-g-MAH/MWCNTs gradient material exhibited a tensile strength of up to 27.3 MPa, while the PA6/POE-g-MAH/BN gradient material showed an even higher tensile strength at 42.91 MPa. Additionally, through three-point bending and interlaminar shear testing, we confirmed that the gradient structural design maintained dimensional stability while preparing PA6 3D-printed materials with excellent mechanical properties. Notably, the BN gradient composites outperformed the MWCNTs gradient materials, with a bending strength of up to 60.91 MPa and an interlaminar shear strength of 10.23 MPa. This approach differs from the traditional method of reducing the crystallinity of blends by incorporating polyolefin elastomers, sacrificing the mechanical properties of PA6, and offers a new method for 3D printing of high-strength, high-dimensional-stability PA6 composites from a structural design perspective. Overall, PA6 composite 3D printing provides a new and effective method.

## Figures and Tables

**Table 1:** The mass fraction of different components.

PA6/wt.%	POE-g-MAH/wt.%	MWCNTs/wt.%	BN/wt.%
93	5.5	2.5	—
90	5	5	—
89	4.5	7.5	
85.5	4.5	10	—
83	4.5	12.5	
81	4	15	—
90	5	—	5
85.5	4.5	—	10
76	4	—	20

**Table 2:** Detailed parameters of S2-FDM 3D printing.

Parameter	Set Value
Nozzle diameter (mm)	0.4
Nozzle temperature (°C)	240
Hot-bed temperature (°C)	30
Gradient configuration A	0
Gradient configuration B	100
Story height (mm)	0.2
Filling rate (%)	100
Print speed (mm s^−1^)	40

**Table 3:** Thermal Property Comparison of the PA6/POE-g-MAH blends.

Name of the Sample	Enthalpy (J/g)	Melting Temperature (°C)	Crystallinity (%)
100PA6	73.63	247.54	38.75
95PA6/5POE-g-MAH	69.17	247.97	38.32
90PA6/10POE-g-MAH	64.74	247.52	37.86
85PA6/15POE-g-MAH	60.13	247.54	37.23

**Table 4:** Thermal Property Comparison of the PA6/POE-g-MAH/MWCNTs blends.

Name of the Sample	Enthalpy (J/g)	Melting Temperature (°C)	Crystallization Temperature (°C)	Crystallinity (%)
PA6/ POE-g-MAH	69.17	247.97	248.53	38.32
PA6/POE-g-MAH/2.5 wt.% MWCNTs	66.86	248.38	244.83	37.83
PA6/POE-g-MAH/5 wt.% MWCNTs	64.04	248.37	244.84	37.45
PA6/POE-g-MAH/7.5 wt.% MWCNTs	62.65	248.39	244.86	37.05
PA6/POE-g-MAH/10 wt.% MWCNTs	59.15	248.38	244.82	36.41
PA6/POE-g-MAH/12.5 wt.% MWCNTs	56.41	248.4	244.83	35.77
PA6/POE-g-MAH/15 wt.% MWCNTs	54.01	248.41	244.81	35.09

**Table 5:** Thermal Property Comparison of the PA6/POE-g-MAH/BN blends.

Sample Name	Enthalpy (J/g)	Melting Temperature (°C)	Crystallization Temperature (°C)	Crystallinity (%)
PA6/ POE-g-MAH	69.17	247.97	248.53	38.32
PA6/POE-g-MAH/5 wt.% BN	64.88	248.48	248.48	37.94
PA6/POE-g-MAH/10 wt.% BN	60.29	248.43	247.73	37.11
PA6/POE-g-MAH/20 wt.% BN	50.93	248.53	247.77	35.27

**Table 6:** Different FDM 3D-printed samples and their numbers.

Number	Typology
A	95PA6/5POE-g-MAH
B	PA6/POE-g-MAH/MWCNTs-COOH (2.5 wt.%)
C	PA6/POE-g-MAH/MWCNTs-COOH (5 wt.%)
D	PA6/POE-g-MAH/MWCNTs-COOH (7.5 wt.%)
E	PA6/POE-g-MAH → POE-g-MAH/MWCNTs-COOH (5 wt.%), from bottom to top
F	PA6/POE-g-MAH → POE-g-MAH/MWCNTs-COOH (10 wt.%), from bottom to top
G	PA6/POE-g-MAH → POE-g-MAH/MWCNTs-COOH (15 wt.%), from bottom to top
H	POE-g-MAH/MWCNTs-COOH (5 wt.%) → PA6/POE-g-MAH, from bottom to top
I	POE-g-MAH/MWCNTs-COOH (10 wt.%) → PA6/POE-g-MAH, from bottom to top
J	POE-g-MAH/MWCNTs-COOH (15 wt.%) → PA6/POE-g-MAH, from bottom to top
K	PA6/POE-g-MAH/BN (5 wt.%)
L	PA6/POE-g-MAH/BN (10 wt.%)
M	PA6/POE-g-MAH → POE-g-MAH/BN (10 wt.%), from bottom to top
N	PA6/POE-g-MAH → POE-g-MAH/BN (20 wt.%), from bottom to top
O	POE-g-MAH/BN (10 wt.%) → PA6/POE-g-MAH, from bottom to top
P	POE-g-MAH/BN (20 wt.%) → PA6/POE-g-MAH, from bottom to top

## Data Availability

Not applicable.
